# Radial Forearm Free Flap for Head and Neck Defect Reconstruction: An Up-to-date Review of the Literature

**DOI:** 10.7759/cureus.35653

**Published:** 2023-03-01

**Authors:** Bader Fatani

**Affiliations:** 1 Dentistry, College of Dentistry, King Saud University, Riyadh, SAU

**Keywords:** defects, maxillofacial, head and neck, reconstruction, radial forearm free flap

## Abstract

The radial forearm free flap has emerged as the mainstay of free flaps for oral cavity soft tissue reconstructions because of its versatility and ability to be used in the reconstruction of large and medium-sized defects. This flap is commonly used to restore head and neck defects, including full-thickness lip and oral cavity defects. This flap offers the opportunity to cover severe defects of the facial region due to its long vascular pedicle and elasticity. In addition to its ease of being harvested, the radial forearm free flap provides a sensate, pliable, and thin skin paddle with a long vascular pedicle. However, it can cause severe morbidity at the donor site, mainly due to exposure of the flexor tendon that results from a failed harvesting of the skin graft, changed sensation in the radial nerve, aesthetic deformity, and reduced range of motion and grip strength. This article aims to review all the up-to-date studies discussing the use of radial forearm free flap in head and neck reconstruction.

## Introduction and background

Reconstruction of the head and neck with microvascular free flaps has been performed since 1959 [1]. In the past, the use of vascularized flaps was considered the most appropriate [2]. Reconstructive methods and techniques have evolved to permit free tissue transfer for the reconstruction of the head and neck [3]. The use of free vascular-supplied tissue, particularly the radial forearm free flap, is considered the last choice of treatment in case of recurrences [2]. Small defects are usually treated with local xenogenic or allogenic grafts. However, in case of extensive defects, dural and osseous structures require a different approach like vascularized tissue transplants [2]. The choice of free flap depends on the tissue type, size, and location of the defect [4,5]. The fasciocutaneous radial forearm free flap became a successful choice for reconstructing defects. This flap gives a wide surface of up to 9 × 12 cm of pliable and thin soft tissue [4]. Previous studies have illustrated the use of radial forearm free flaps in different surgeries [1-4]. However, few up-to-date studies have discussed the use of this flap in head and neck reconstruction. This article aims to review all the up-to-date studies discussing the use of radial forearm free flap in head and neck reconstruction.

## Review

Methods

This review article involved an evaluation of published studies discussing the use of radial forearm free flap in head and neck reconstruction. The population, intervention, control, and outcomes (PICO) framework was used as follows: in patients with head and neck defects, is radial forearm free flap compared to other methods effective in head and neck reconstruction? 

Three databases, PubMed, Web of Science, and Google Scholar, were used to gather the most relevant and up-to-date papers discussing this topic. Studies were searched from the year 2019 to 2023. A search set combined a range of keywords such as "radial forearm free flap", "defect reconstruction", and "head and neck reconstruction". By using this method, studies discussing the use of radial forearm free flap in head and neck reconstruction were obtained. We included all the available, relevant, and up-to-date studies discussing radial forearm free flap, surgical approach, survival rate, and complications in the inclusion criteria. The studies that were outdated or had poor methodological quality and insufficient data were excluded. Primary outcomes were determined if the studies discussed the use of radial forearm flaps in clinical studies. On other hand, all the non-clinical studies were chosen as secondary outcomes. The initial screening revealed 129 studies. After applying our inclusion criteria, the most relevant studies were selected and used in the current review. This review was conducted by reviewing 24 studies related to the use of radial forearm free flap in head and neck reconstruction.

Radial forearm free flap

The radial forearm free flap was first described in 1981 [1,6]. This flap offers the opportunity to cover severe defects of the facial region due to its long vascular pedicle and elasticity. However, it lacks the rigid factor for nasal framework reconstruction [7]. The radial forearm free flap has emerged as the mainstay of free flaps for oral cavity soft tissue reconstructions because of its versatility and ability to reconstruct large and medium size defects [3]. This flap is commonly used in the restoration of head and neck defects, including full-thickness lip and oral cavity defects [6]. Yet, their main disadvantage is the clear forearm scar that remains after the procedure. Typically, the defect in the skin is treated by full-thickness grafting or split-thickness grafting, however, a possible outcome is severe morbidity at the donor site area as well as poor aesthetic outcome, restriction in the function of the forearm, and healing problems [8].

**Figure 1:**
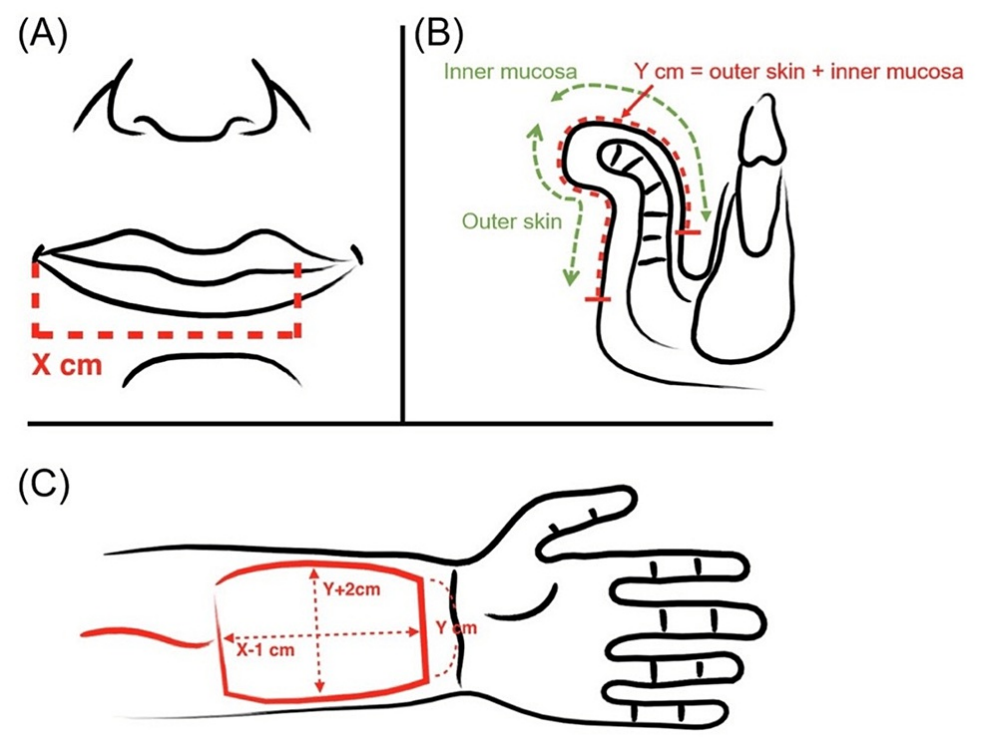
Barrel-shaped design of the radial forearm free flap. (A) In case the horizontal defect is “X” cm; (B) “Y” cm is the vertical defect. (C) The flap is picked with a dimension of the transverse dimension of Y + 2 cm and X − 1 cm. © Lin et al. [10] 2020. Open Access. This article is licensed under a Creative Commons Attribution 4.0 International License, which permits use, sharing, adaptation, distribution, and reproduction in any medium or format, as long as you give appropriate credit to the original author(s) and the source, provide a link to the Creative Commons licensce, and indicate if changes were made.

**Figure 2:**
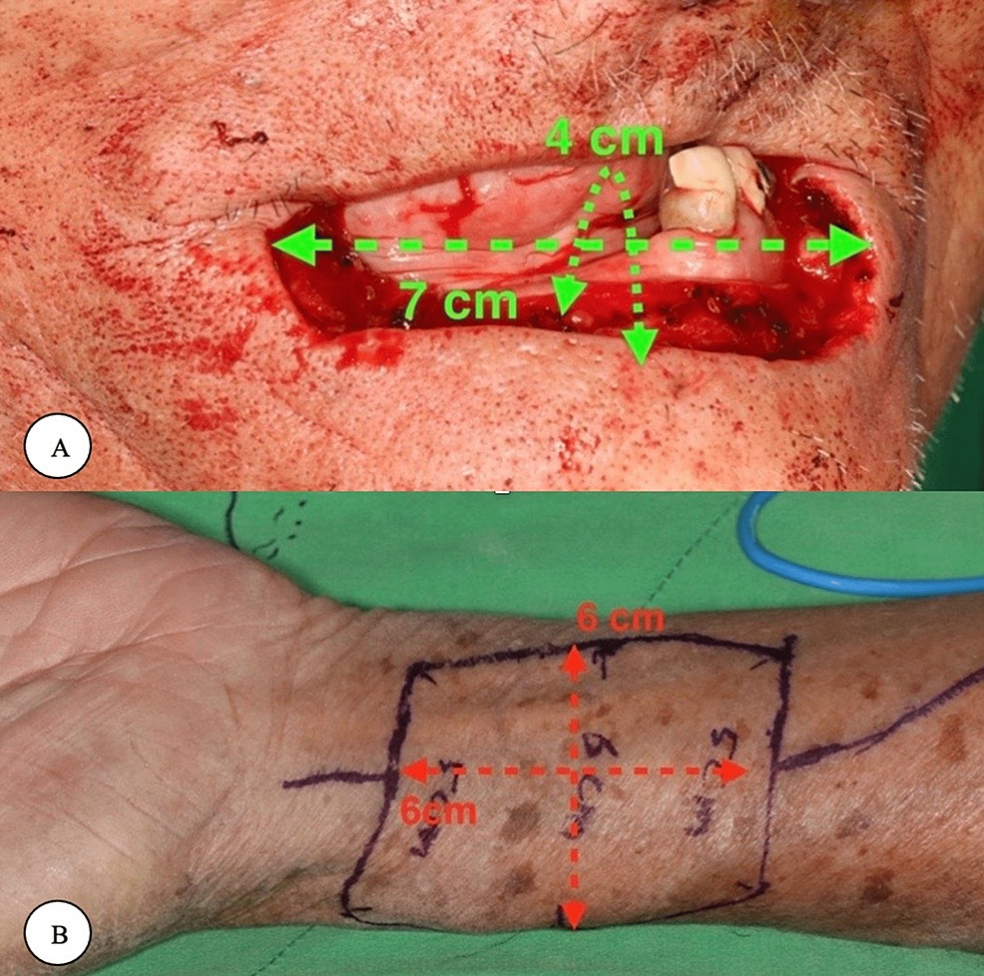
(A) the vertical defect is 4 cm, and the horizontal defect is 7 cm. (B) The free forearm flap was harvested with a 6 × 6 cm dimension. © Lin et al. [10] 2020. Open Access This article is licensed under a Creative Commons Attribution 4.0 International License, which permits use, sharing, adaptation, distribution, and reproduction in any medium or format, as long as you give appropriate credit to the original author(s) and the source, provide a link to the Creative Commons license, and indicate if changes were made.

The radial forearm free flap provides a sensate, pliable, and thin skin paddle with a lengthy vascular pedicle and is easy to harvest technically. However, it can cause severe morbidity at the donor site, mainly from exposure of the flexor tendon that results from a failed harvesting of the skin graft, changed sensation in the radial nerve, aesthetic deformity, and reduction in the range of motion and grip strength [6]. The lateral arm flap is considered a distinctive and highly versatile soft tissue flap. The versatility of the flap provides a continuous alteration of the flap, in addition to its uses in different sites and forms with outstanding contour results [9]. A modified suprafascial technique can provide less risk of exposure of the flexor tendon by aiming to preserve the deep area of the fascia that overlays the tendons, therefore improving the graft recipient bed [6]. The reconstruction of defects using osteocutaneous radial forearm free flap is indicated when a rigid support system is required, particularly with postoperative radiation or for big bony defects of the skull base in case no other different choice is available. However, multiple considerations should be acknowledged about this procedure [2]. This procedure is advocated to be performed by a multidisciplinary team. The radial forearm free flap is related to common postoperative complications, in addition to significant morbidity [2]. The radial forearm free flap has some disadvantages and can be prevented by using the ulnar forearm fasciocutaneous flap. This flap was introduced in 1984 by Lovie et al [6]. It provides similar advantages to the radial flap. However, it has less donor site morbidity and superior tissue characteristics. The sensation of the ulnar nerve of the ulnar forearm fasciocutaneous flap is typically not affected, the skin of the flap is less hairy than the regular radial flap skin, and the location of the skin paddle results in fewer donor site defects. The ulnar forearm flap is rarely used due to the ongoing belief that it is difficult to harvest these flaps and that the ulnar artery is essential for hand circulation [6]. The Allen test is a test that is most often used for evaluation and assessment of the contributions of the ulnar and radial arteries to the perfusion by the palmar arches [6]. This test is considered unreliable and subjective in nature. Moreover, the sensitivity is 91.7% and its specificity is 54.5% [6]. Most surgeons nowadays use this test with a variety of techniques such as intraoperative testing and pulse oximetry to improve the problems associated with the Allen test [6]. The radial artery carries the main vascular supply to the hand in association with a previous result of a study that showed that the radial artery is smaller than the ulnar artery in the proximal forearm. However, the radial artery is shown to be a larger diameter at the wrist [6]. A demonstration of the barrel-shaped design of the radial forearm free flap and flap size is shown in Figures 1-2.

Surgical approach

**Figure 3:**
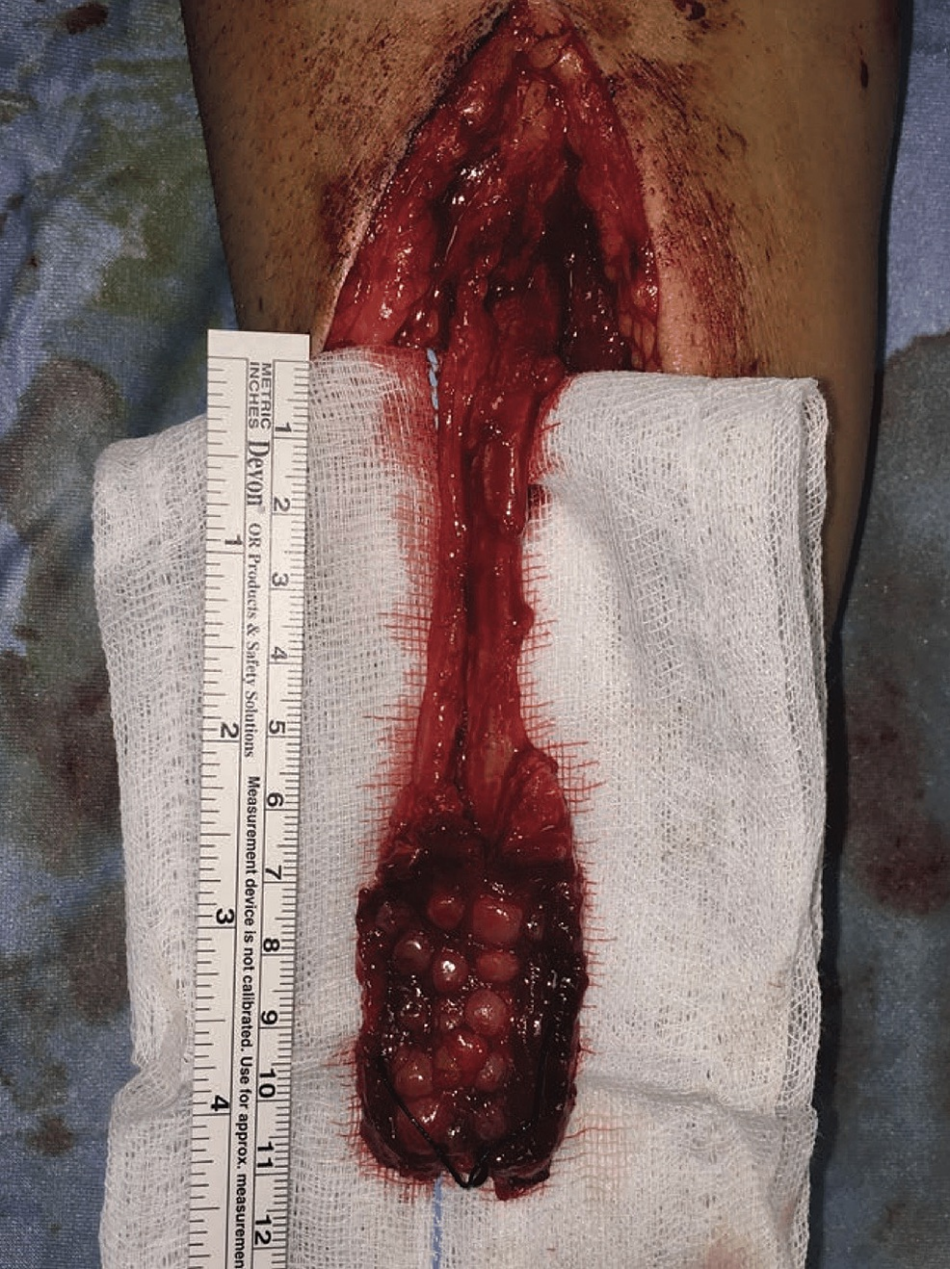
Buccal mucosa grafts morselized in the radial forearm free flap before tongue reconstruction. © De Santis et al. [16] 2020. This is an open-access article distributed under the terms of the Creative Commons Attribution-Non Commercial-No Derivatives License 4.0 (CCBY-NC-ND), where it is permissible to download and share the work provided it is properly cited. The work cannot be changed in any way or used commercially without permission from the journal.

While the radial forearm free flap provides a lot of advantages, this flap also has several problems [4]. Transfer of free tissue needs advanced experience, training, and surgical skills [4]. Moreover, this procedure requires specific equipment including microsurgical instruments and an operating microscope and is often lengthy and time-consuming to perform [4]. The blood supply to the forearm is somewhat variable and depends mainly on the brachial artery; this artery separates into the radial and ulnar arteries [6]. When entering the hand, the ulnar artery is superficial to the flexor retinaculum which provides the superficial palmar arch [6]. A new approach for radial forearm free flap is the shape-modified technique [8]. This technique achieves the primary closure of the defect which helps avoid skin grafts [8]. This method is based on the design of narrow flap strips depending on the cutaneous perforators that cross along the path of the radial artery [8]. These strips can be divided and folded as desired to suit the defect and get an adequate width [8]. Balakrishnan et al. aimed to assess the efficacy and the outcomes of a single-stage reconstruction technique of composite post-excisional commissure and pericommissural defects with a combination of Pacman-style free radial forearm flap and oblique elastic musculomucosal flap [11]. The author suggested that the combination of these flaps for the composite post-excisional commissure and pericommissural defects in single-stage reconstruction is a valuable supplement to re-establish the continence and the aesthesis for the reconstructed site [11]. On other hand, Piotrowska et al. presented a combined flap of radial forearm free flap and auricular free flap for the reconstruction of nasal defects [7]. The author reported that the combination of these flaps gave a satisfactory aesthetic outcome and appears to be optimal for the reconstruction of extended nasal defects [7]. Wu et al. inspected the feasibility of glossectomy defect reconstruction using the radial forearm free flap without a tracheostomy tube [12]. The findings suggest that glossectomy defect reconstruction using the radial forearm free flap without a tracheostomy tube is feasible among patients with unilateral neck dissection and small tongue base defects [12]. Poissonnet et al explained the surgical technique for reconstruction of tracheoesophageal fistula with pharyngoesophageal stenosis using a double skin paddle fasciocutaneous radial forearm free flap [13]. The main technique key points include correcting the position of the two skin paddles to reconstruct the posterior tracheal wall and anterior pharyngoesophageal wall, in addition to de-epidermization of the middle region of the flap that will be positioned in the tracheoesophageal space [13]. Moreover, Pipkorn et al. study reported that delivering the endoscopic adipofascial radial forearm free flap to the skull base by a transbuccal corridor or Caldwell-Luc is a feasible choice with low morbidity and high success in case other reconstructive options failed [14]. A comparison of the clinical application results of the radial forearm free flap and free posterior tibial artery perforator flap for reconstruction of facial defects was evaluated by Wang et al [15]. The author illustrated that a free posterior tibial artery perforator flap can be considered an alternative to the radial forearm free flap in head and neck reconstruction with its clinical and aesthetic advantages [15]. A prospective study by Chavre et al. compared the donor site morbidity between a new shape-modified technique and the conventional technique based on the quality of life, functional outcomes, and aesthetics [8]. The author explained that the shape-modified technique for the radial forearm flap offers primary closure at the donor area with good healing [8]. The aesthetic and functional results at the donor area were better in the shape-modified technique. The new approach is suggested to be suitable in case the defect was small in size [8]. De Santis et al. study suggested that a prelaminated fasciomucosal radial forearm free flap is effective for the tip of the tongue reconstruction, particularly in specific cases where articulation and speech intelligibility are important [16]. In addition, Rahman et al. stated that the composite radial forearm flap with palmaris longus tendon is considered a reliable and good choice for total lip reconstruction [17]. On other hand, Kitamura et al. illustrated that the combined use of labia minora peripheral skin graft and an innervated free radial forearm flap could be an option for the reconstruction of extensive defects in the upper lip [18]. Moreover, Maruccia et al. suggested that a prelaminated radial forearm free flap could be a great reconstructive choice in large defects of the columella [19]. Lin et al. study also showed that a barrel-shaped design free forearm flap exhibited better results than a rectangular design for lower lip reconstruction based on social contact and function [10]. Demonstration of buccal mucosa grafts morselized in the radial forearm free flap before tongue reconstruction is shown in Figure 3.

Complication and survival rate

**Figure 4:**
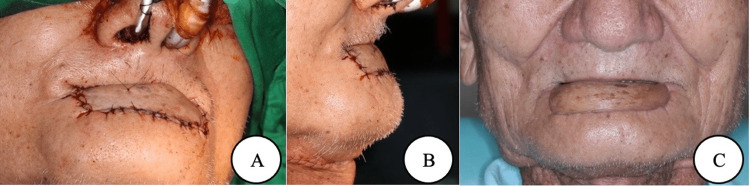
Demonstration of postoperative view (a) lateral view (b), the middle portion of the flap is convex slightly to ensure the restoration of oral competence. (c) One-year follow-up. © Lin et al. [10] 2020. Open Access This article is licensed under a Creative Commons Attribution 4.0 International License, which permits use, sharing, adaptation, distribution, and reproduction in any medium or format, as long as you give appropriate credit to the original author(s) and the source, provide a link to the Creative Commons license, and indicate if changes were made.

The radial forearm free flap carries some harmful complications such as thenar hypoesthesia, wound infection, and hematoma [4]. Most of these flaps will require donor site coverage in the form of a split-thickness graft; this in turn has more complications including tendon exposure and skin graft loss that could cause further wrist dysfunction [4]. Donor site morbidity of the radial forearm free flap and posterior tibial artery perforator flap after cancer ablation was evaluated by Mai et al [20]. The findings of the study suggested no significant difference between the preoperative and postoperative ranges of wrist and ankle movements [20]. Moreover, Kim et al. suggested that a strip design can facilitate the simple creation of externalized monitoring flap with a buried radial forearm free flap with no additional morbidity at the donor site [21]. Ibrahim et al. compared the outcome of using radial forearm free flaps and facial artery musculomucosal flaps for the reconstruction of medium-size defects in the oral cavity, largely focusing on functional and surgical outcomes [3]. The findings of this study showed that the facial artery musculomucosal flap can be used to reconstruct medium-size defects with similar functional outcomes to the radial forearm free flaps, in addition to decreasing both the morbidity and costs of the procedure [3]. In addition, a case report by Bitner et al. showed a rare head and neck chondrosarcoma that developed from the mandibular coronoid process which was reconstructed using a custom polyetheretherketone implant; the contour was restored with a radial forearm free flap [22]. Ranganath et al. study aimed to compare aesthetics, function, surgical morbidity, and health-related quality of life among patients treated with radial forearm free flap and anterolateral thigh free flap for intra-oral reconstruction [23]. The study explained that the anterolateral thigh free flap achieved less donor site morbidity, equal oral function, and survival rates compared to the radial forearm free flap [23]. A study by Gschossmann et al. discussed the success rates and feasibility of contralateral anastomosis in free flap reconstruction [1]. The author concluded that contralateral anastomosis is a safe and successful choice in the reconstruction of head and neck defects, particularly with using a radial forearm free flap [1]. Krane et al. compared the aesthetic and morbidity results of split-thickness skin grafts and full-thickness skin grafts in the reconstruction of the donor site of the forearm free flap [24]. The author suggested that using full-thickness skin grafts in the reconstruction of the donor site of the forearm free flap would give a better aesthetic outcome without the morbidity of the donor site or wound creation compared to split-thickness skin grafts [24]. Gur et al. compared current free flap options for intraoral lining and tongue reconstruction. The author suggested that the anterolateral thigh flap is considered the best choice in an average-weight person due to its pliability, reliability, and constant vascular structure [25]. Demonstration of the postoperative view is shown in Figure 4.

**Table 1:** Summary of relevant studies discussing the radial forearm free flap.

Author	Year of publication	Objective	Outcome
Balakrishnan et al.	2022	Assessment of the efficacy and the outcomes of a single-stage reconstruction technique of composite post-excisional commissure and pericommissural defects with a combination of Pacman-style free radial forearm flap and oblique elastic musculomucosal flap	The combination of these flaps for the composite post-excisional commissure and pericommissural defects single-stage reconstruction is a valuable supplement to re-establish the continence and the aesthesis for the reconstructed site
Wu et al.	2022	Assessment of the feasibility of glossectomy defects reconstruction using the radial forearm free flap without a tracheostomy tube	Glossectomy defect reconstruction using the radial forearm free flap without a tracheostomy tube is feasible among patients with unilateral neck dissection and small tongue base defects
Wang et al.	2021	Comparison of the clinical application results of the radial forearm free flap and free posterior tibial artery perforator flap for reconstruction of facial defects	Free posterior tibial artery perforator flap can be considered an alternative to the radial forearm free flap in head and neck reconstruction with its clinical and aesthetic advantage
Chavre et al.	2020	Comparison of the donor site morbidity between a new shape-modified technique and the conventional technique based on the quality of life, functional outcomes, and esthetics	The shape-modified technique for the radial forearm flap offers primary closure at the donor area with good healing. The esthetic and functional results at the donor area were better in the shape-modified technique. The new approach is suggested to be suitable in case the defect was small in size
Mai et al.	2022	Assess donor site morbidity of the radial forearm free flap and posterior tibial artery perforator flap after cancer ablatio	No significant difference between the preoperative and postoperative ranges of wrist and ankle movement
Ibrahim et al.	2021	Comparison of the outcome of using radial forearm free flaps and facial artery musculomucosal flaps for the reconstruction of medium-size defects in the oral cavity; the author focused on functional and surgical outcomes	The facial artery musculomucosal flap can reconstruct medium-size defects with similar functional outcomes to the radial forearm free flaps, in addition to decreasing both the morbidity and costs of the procedure
Ranganath et al.	2022	Comparison of aesthetics, function, surgical morbidity, and health-related quality of life among patients treated with radial forearm free flap and anterolateral thigh free flap for intra-oral reconstruction	The anterolateral thigh free achieved less donor site morbidity, equal oral function, and survival rates compared to the radial forearm free flap
Gschossmann et al.	2022	Evaluation of the success rates and feasibility of contralateral anastomosis in free flap reconstruction	Contralateral anastomosis is a safe and successful choice in the reconstruction of head and neck defects, particularly with using a radial forearm free flap
Krane et al.	2020	Comparison of aesthetic and morbidity results of split-thickness skin grafts and full-thickness skin grafts in the reconstruction of the donor site of the forearm free flap	Full-thickness skin grafts in the reconstruction of the donor site of the forearm free flap gave a better aesthetic outcome without the morbidity of the donor site or wound creation compared to split-thickness skin grafts
Gur et al.	2022	Comparison of current free flap options for intraoral lining and tongue reconstruction	The anterolateral thigh flap is considered the best choice in an average-weight person due to its pliability, reliability, and constant vascular structure

## Conclusions

The radial forearm free flap shows great value in head and neck reconstructions due to its versatility and ability to reconstruct large and medium size defects. However, some points need to be considered by the practicing surgeon. Usage of radial forearm free flap is associated with common postoperative complications and significant morbidity to the surgical procedure. Most of the discussed studies did not demonstrate significant postoperative complications regarding the flap. However, careful examination and treatment planning should be done by the practicing surgeon to avoid any unanticipated complications.
